# Erratum for “Urinary cortisol‐creatinine ratio in dogs with hypoadrenocorticism”

**DOI:** 10.1111/jvim.16731

**Published:** 2023-04-21

First published: 11 February 2022 https://onlinelibrary.wiley.com/doi/10.1111/jvim.16358; Volume 36, Issue 2.

The urinary cortisol : creatinine ratio (UCCR) was erroneously calculated by dividing the urine cortisol concentration in μg/dL by the urine creatinine concentration in mg/dL, without converting the 2 analytes into the same measurement unit. To provide the correct values of UCCR, the UCCR was calculated again by converting cortisol and creatinine in the same measurement unit (nmol/L).

Section 3 Analytical Procedures, first paragraph, last sentence, should be as follows. “The UCCR was calculated by dividing the urine cortisol concentration (nmol/L) by the urine creatinine concentration (mmol/L).”

Reference 29 is removed from the References list.

Section 4 Results, UCCR subsection, first sentence should be as follows. “The median UCCR was 2.03 × 10^−6^ (1.04‐3.81 × 10^−6^), 32.09 × 10^−6^ (7.68‐245.5 × 10^−6^), and 10.55 × 10^−6^ (3.47‐54.05 × 10^−6^) in dogs with HA, dogs with DMHA and healthy dogs, respectively.”

Section 4 Results, UCCR subsection, fifth sentence should be as follows. “The median UCCR in dogs with DMHA and BSC ≤2 μg/dL (≤55 nmol/L) was 27.14 × 10^−6^ (7.68‐245.46 × 10^−6^).

Section 4 Results, UCCR subsection, last sentence should be as follows. “A cut‐off value of UCCR <4.4 revealed 100% sensitivity (95% CI: 69.1‐100) and 97.3% specificity (95% CI: 85.8‐99.9) in diagnosing HA.”

Figure 1, to be replaced with this new Figure 1.

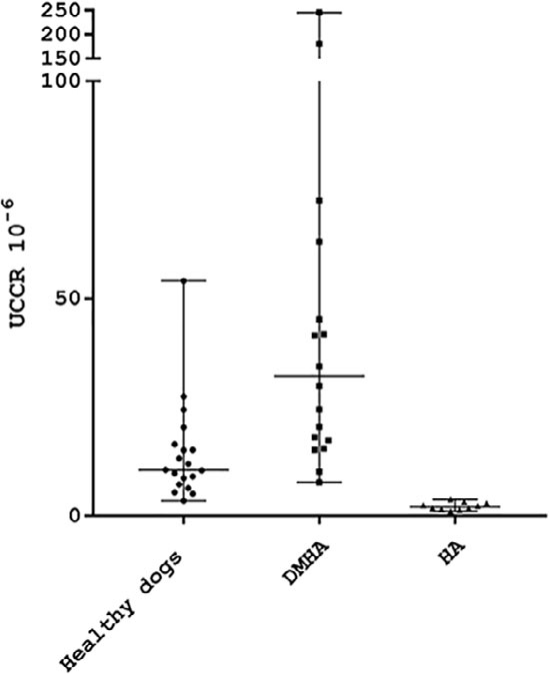

**FIGURE 1** Scatter scale plot comparing urinary corticoid: creatinine ratio (UCCR) of dogs with hypoadrenocorticism (HA, n = 10), dogs with disease mimicking hypoadrenocorticism (DMHA, n = 18) and healthy dogs (healthy, n = 19). The horizontal bars represent the median, the maximum, and the minimum value of each group.

